# 

**Published:** 1892-05-07

**Authors:** 


**Notices of Births, Deaths, and Marriages,** are inserted in this column at a charge of 2s. 6d. for an announcement not exceeding four lines.

### NOTICE TO CORRESPONDENTS.

All MS., Letters, Books for Review, and other matters intended for the Editor should be addressed The Editor, The Lodge, Porchester Square, London, W.

The Editor cannot undertake to return rejected M.S., even when accompanied by stamped directed envelope.

**Notice to Advertisers and Subscribers.**

All Advertisements, Orders for Copies of The Hospital, and Business Communications should be addressed to The Publisher, *not to the Editor*, The Hospital, 140, Strand, W.C.

The Cost of Subscription, post free, is as follows:—Per week, 2½d.; per quarter, 2s. 6d.; per half-year, 5s.; per annum, 8s. 6d. Foreign:—Per annum, 10s. 8d.; payable, in all cases, in advance.

**"Burdett's Hospital Annual," 1891—1892.**

Third year of publication. 600 pp. Boards, gilt lettering. A most complete guide to British and Colonial Hospitals, Dispensaries, Nursing and Convalescent Institutions, and Asylums. Price 3s. 6d. Published by The Scientific Press (Limited), 140, Strand, W.C.

**NOTICE.—The Index for Vol. X. (ending September, 1891), is on sale at the Office of this Journal, price 2½d., post free.**

### Scraps and Gleanings.

At the Glasgow Hospital for Skin Diseases, 1,385 cases were treated during the past year.

At a meeting held recently, it was proposed to build a joint isolation hospital for Guildford and Godalming.

Baron De Hirsch has forwarded a donation of £700 to the East London Hospital for Children, Shadwell.

The Hospital Ball at Ipswich was a grand success. The dance music was supplied by the Suffolk Artillery Militia, and about 150 guests were present.

Cumberland and Westmoreland have appointed May 8th to be observed as Hospital Sunday this year. The amount realised last year was £893 16s.

A concert, under the direction of Mr. Sandor Marei, will be given on Tuesday evening next at 74, South Audley Street, in aid of the Working Girls' Club at Ipswich.

At a special Court of Governors of the East Suffolk and Ipswich Hospital, held at that institution, it was proposed to erect a separate building for the nursing staff, at a cost of £1,600.

At the anniversary festival of the British Orphan Asylum, held at the Hotel Metropole, subscriptions amounting to about £2,100 were announced in aid of the funds of the institution.

At the annual general meeting of the supporters of the Asylum for Idiots, Earlswood, Redhill, it was reported that scientific modes of training had been tried, both physical and mental, with signal success.

The Sub-Committee of the Infectious Diseases Hospital, Yarmouth, have decided that more accommodation is necessary. Eight additional beds are to be added, making, with the present twelve beds, twenty in all.

An outbreak of small-pox has occurred at Penge. Six cases have been reported to the District Medical Officer. Of this number two cases have proved fatal. Neither of the patients who had died had been vaccinated.

A series of ambulance lectures were given last year to a large number of ladies by the Matron and doctors of the Barrington Hospital, Limerick. The proceeds of these lectures realised £22 for the funds of the hospital.

Mrs. Eliza Allison has died at Chapel House, Richmond, at the age of 101. She enjoyed good health till within a short time of her death. Her husband, who has been dead some years, fought in the Battle of Waterloo.

Dr. Chadwick who for six years was attached to the London Hospital, and Dr. Griffith, who in 1887 became professor of anatomy at the Yorkshire College, have been elected honorary assistant physicians to the Leeds General Infirmary.

At the annual meeting of the subscribers to the Surbiton Cottage Hospital, it was stated that 151 patients were admitted during the year. The accounts showed a balance of £31 9s. 7d. against the hospital. The sum of £61 19s. 4d. was collected in the money-boxes.

Dr. Thomas B. Diplock, Coroner for West Middlesex and the Western Division of London, died at his residence at Chiswick, April 29th, from cancer of the tongue, after an illness of eight months' duration. The deceased was born in 1830, and was elected Coroner in May, 1868.

The annual report of the Adelaide Hospital, Dublin, states that the increased accommodation has proved how much it was needed by the fact that 77 more intern patients were treated during the past year than in 1891. Plans for the convalescent home in connection with the hospital are being prepared.

Towards the new Hospital Fund of St. Mary's Hospital, Manchester, £34,082 has already been subscribed. For seven weeks last summer the present hospital was closed in order that the reconstruction of the drainage, which was entrusted to Mr. J. Corbet, C.E., might be carried out, at a cost of £600.

The Very Rev. the Dean of Worcester (Dr. Forrest), preaching at St. Peter's, Cranley Gardens, on behalf of the Brompton Consumption Hospital, said the income required was about £25,000, towards which the only fixed revenue was about £5,000. There were 321 beds in the two buildings, connected by a subway. The collection amounted to over £80.

The sale of work for the clearing off the debt of £300, incurred in the building of a new wing to the Boscombe Infirmary and Cottage Hospital, was highly satisfactory, the whole amount being realised in the two days' sale. In addition to this sum the offertory in St. John's Church on Good Friday morning for the hospital, amounted to £11 15s. 8d., and a special donation of £5 from Miss Banks was also received.

The total receipts of the Carmarthenshire Infirmary for 1891 amounted to £1,511 4s. 11d., and the expenditure to £1,641 10s. 5d., leaving an adverse balance of £130 5s. 6d. Several improvements were carried out, amongst them an accident ward has been completely furnished with new and improved appliances. New beds have replaced the old ones, which for some time had been condemned as unfit for infirmary use.

A meeting of the Executive Committee of the National Leprosy Fund was held recently, in the Conference Room of the House of Commons. A memorandum with regard to the report of the three Commissioners as to their investigations in India concerning leprosy was submitted. It is to be attached to the report of the Commissioners, which will be issued so soon as the printed copies are received from India.

By desire of Princess Christian of Schleswig-Holstein, a series of nine *tableaux vivants* has been arranged by the Countess of Cottenham, Mrs. Tyssen-Amherst, Lady William Cecil, and her sisters, under the direction of Mr. Hermann Schmiechen, for the benefit of the Royal School of Art Needlework. The *tableaux* will represent the history of art needlework. Queen's Gate Hall has been chosen for the performances, which will take place on the 19th and 20th of May.

### THE STUDENT OF MEDICINE.

[All communications for this Supplement should be addressed to The Editor of The Hospital, at 140, Strand, with the words, "Students' Column," written in the left hand top corner of the envelope.]

### STUDENTS' BOOKSHELF.

### CAMPING OUT*.

Medical students have, as a rule, but few opportunities of indulging in extensive holidays, so that it is a matter of considerable importance to them that they should derive from them as much pleasure and health as is compatible with the short time at their command. We should therefore recommend all medical students, before they decide how to spend their summer holidays, to read Mr. Macdonell's new contribution to the "All-England Series" on the subject of camping out. It is a little book full of fascinating suggestions, and is a complete guide to all who care to indulge in this most delightful form of amusement; it gives one the choice of a large variety of routes, and tells one exactly what impedimenta to take in order that the trip may be thoroughly comfortable or even luxurious, and what is quite as important, it gives one all the necessary information for procuring them; and last, but not least, it instructs the tourist as to what to avoid, what to eat, and how to cook it.

The author is himself an old hand at this sort of thing, and he can give the reader any amount of "tips," especially with regard to boating expeditions; and, as an old rowing man, he naturally gives the preference to tours by water, and from practical experience in expeditions by land, in boats, in caravans, and on foot, we are inclined to agree with him when he states that, "Of all ways of enjoying a holiday, a boating expedition has more to recommend it than any other." Our one regret is that Mr. Macdonell's book was not published sooner, so that we might not have fallen into those many pitfalls and mistakes which inevitably attend the first experience of the amateur camper.

From a hygienic point of view, Mr. Macdonell enlarges on the advantages of this form of expedition in the following words: "The benefit to health accruing from an absolutely out-door life, extending over several weeks, especially when combined with the large amount of physical exercise taken during a boating tour, is obvious to everyone. I myself have invariably returned from such trips in a state of health which may be described as absolutely perfect, and have always been complimented by friends as to my peculiarly robust appearance on such occasions; in fact, if the sum total of years which each expedition had, according to their statements, apparently taken off my age were actually substracted therefrom, I should not yet be born for some years to come." We cannot, however, agree with Mr. Macdonell that a night spent "al fresco," enveloped in river mist, is unattended with risk, although we can readily believe that an iron constitution, such as he is possessed of, need not necessarily suffer detriment; the more equable temperature of a tent is more to our taste, both from a hygienic and practical point of view, and as a journal which professes to teach the rules of health, we feel it is our duty to warn campaigners against making the dangerous experiment which Mr. Macdonell can, it seems, perform with comparative impunity.

With regard to the expenses of such expeditions, on reference to the chapter devoted to this subject, the reader will be delighted at their comparative lightness. Speaking

* "Camping Out." The "All-England" Series. By A. A. Macdonell.

THE STUDENT OF MEDICINE—(*continued*).

roughly, about £2 15s. will cover the total cost of a week's camping out, provided that the expedition lasts for two weeks and upwards.

Medical students are not, as a rule, over-fastidious; but we are not prepared to state that they would entirely approve of the following recommendation of the author: "It will, in the first place, be a great convenience to have as common property in camp-life a folding indiarubber bath . . . it will often be a boon in foreign hotels. Its main use, however, is to do service in camp, not only for a morning tub on occasions when the river does not happen to be suitable for bathing, but also for washing one's face and hands in; it is at the same time very convenient for cleaning plates and spoons after a meal."

### HOSPITAL NEWS.

The West End Hospital for Diseases of the Nervous System, Paralysis, and Epilepsy was reopened on April 11th, with the following staff:—Physicians: Drs. A. Hughes Bennett, T. Outterson Wood, W. Wallis Ord, S. H. Armstrong, Edward Squire, and Armand de Watteville. Surgeon: Mr. Edward Cotterell. Ophthalmic Surgeon: Mr. H. Frank Dodd. Throat and Ear Surgeon: Dr. Dundas Grant. Dental Surgeon: Mr. C. Vincent Cotterell.

Guy's Hospital.—The current number of the *Gazette* contains the notes of a lecture by Dr. Hall White, on "Headache."

### APPOINTMENTS.

Bain, J. G., M.B., C.M., appointed House Surgeon to the Taunton and Somerset Hospital. Byrne, R. P., B.A., R.U.I., L.R.C.P.Edin., appointed House Surgeon to the North Charitable Infirmary, Cork. Chadwick, C. M., M.A., M.D.Oxon., M.R.C.P.Lond., appointed Honorary Assistant Physician, to the Leeds General Infirmary. Clemons, G. E., M.B., C.M.Edin., appointed House Surgeon to the Infirmary for Children, Liverpool. Cotterell, E., L.R.C.P.Lond., F.R.C.S., appointed House Surgeon to the Out-patients, London Lock Hospital. Fraser, J., appointed Assistant Medical Officer at the Central London Sick Asylum. Griffith, T. W., M.D., M.B., C.M.Aberd., appointed Honorary Assistant Physician to the Leeds General Infirmary. Herschell, G., M.D.Lond., M.R.C.S., appointed Physician to the National Hospital for Diseases of the Heart, Soho Square, W. Lane, J. E., F.R.C.S., appointed Surgeon to the In-patients at the London Lock Hospital. Mort, H. B., L.R.C.P.Lond., M.R.C.S.Eng., appointed Honorary Physician to the Governesses' Home, Southport. Passmore, W. E., L.S.A., appointed Assistant Medical Officer to the Wandsworth and Clapham Infirmary. Samuelson, G. S., M.B.Edin., appointed Medical Officer to the Gundagai District Hospital, N.S.W. Shaw, H., M.B., M.A.Cantab., appointed Assistant House Surgeon to the Derbyshire Royal Infirmary.

### VACANCIES.

### Resident Medical Officers.

The following vacancies are announced this week, the amount of salary in each case being given after the name of the institution:

Chelsea Hospital for Women, Fulham Road, S.W., Clinical Assistant.

General Hospital, Nottingham, Assistant House Physician—Board, &c.

Manchester Hospital for Consumption and Diseases of the Chest and Throat, Resident Medical Officer—Salary £60 per annum, with board, &c.

Rotherham Hospital, Rotherham, Assistant House Surgeon—Rooms, &c. Appointment for six months.

Royal Surrey County Hospital, Guildford, Clinical Assistant—Board, &c.

University College Hospital, London, Resident Medical Officer.

### Non-Resident Medical Officers.

Ballaculish Slate Works, Ballaculish, Medical Officer; unmarried—Salary £225 per annum, and general practice.

Charing Cross Hospital Medical School, Lecturer on Biology.

Hospital for Sick Children, Great Ormond Street, Bloomsbury, W.C., Medical Registrar and Pathologist—Appointment for one year. Honorarium, 50 guineas at end of term.

King's College, London, Curator of the Museum.

Northampton Friendly Societies' Medical Institute, Assistant Medical Officer; outdoor—Salary £[illegible]0 per annum.

St. John's Hospital for Diseases of the Skin, Leicester Square, Honorary Assistant Medical Officer to the Convalescent Branch, Bellgarth, Temple Fortune, Finchley Road, N.W.

